# Evaluation of the Toxicity of Bisphenol A in Reproduction and Its Effect on Fertility and Embryonic Development in the Zebrafish (*Danio rerio*)

**DOI:** 10.3390/ijerph19020962

**Published:** 2022-01-15

**Authors:** Lilian de Paula Gonçalves Reis, Antonio Jesús Lora-Benítez, Ana Mª Molina-López, Rafael Mora-Medina, Nahúm Ayala-Soldado, Mª del Rosario Moyano-Salvago

**Affiliations:** Department of Anatomy and Comparative Pathology and Toxicology, Faculty of Veterinary Medicine, Campus de Rabanales, University of Córdoba, Darwin Building, 14071 Cordova, Spain; v22gorel@uco.es (L.d.P.G.R.); v12lobea@uco.es (A.J.L.-B.); v02momer@uco.es (R.M.-M.); r.moyano@uco.es (M.d.R.M.-S.)

**Keywords:** bisphenol A, zebrafish, fertility, embryotoxicity, endocrine-disrupting chemical

## Abstract

Bisphenol A (BPA) is a chemical substance commonly used in the manufacture of plastic products. Its inhalation or ingestion from particles in suspension, water, and/or polluted foods can trigger toxic effects related to endocrine disruption, resulting in hormonal, reproduction, and immunological alterations in humans and animals. The zebrafish (*Danio rerio*) is an ideal experimental model frequently used in toxicity studies. In order to assess the toxic effects of BPA on reproduction and embryonic development in one generation after parental exposure to it, a total of 80 zebrafish, males and females, divided into four groups in duplicate (*n* = 20) were exposed to BPA concentrations of 500, 50, and 5 µg L^−1^, along with a control group. The fish were kept in reproduction aquariums for 21 days. The embryos obtained in the crosses were incubated in a BPA-free medium and observed for signs of embryotoxicity. A histopathological study (under optical and electron microscopes) was performed of adult fish gonads. The embryos of reproducers exposed to BPA were those most frequently presenting signs of embryotoxicity, such as mortality and cardiac and musculoskeletal malformations. In the histopathological studies of adult individuals, alterations were found in ovocyte maturation and in spermatazoid formation in the groups exposed to the chemical. Those alterations were directly related to BPA action, affecting fertility in both sexes, as well as the viability of their offspring, proportionally to the BPA levels to which they were exposed, so that our results provide more information by associating toxic effects on the offspring and on the next generation.

## 1. Introduction

Bisphenol A (BPA) is a synthetic organic compound used in the synthesis of carbonates, epoxy resins, and thermal paper [1,2]. It gives polycarbonate plastics, for example, a greater resistance at high and low temperatures, hardness, tenacity, and resistance to many acids and oils. Plastic production in the world and in the European Union in 2019 was of 368 and 57.9 million tons, respectively, with a turnover of 350,000 million euros in the same year [3]. The global demand for BPA was 7000.9 Kilo tons in 2013, reaching 9618.7 Kilo tons by 2020 and increasing at a compound annual growth rate of 4.7% from 2014 to 2020 [4].

BPA’s use in various commonly consumed goods, such as bottles, food containers, etc. could lead to the pollution of food, the environment, humans, and animals. Exposure can occur through oral, inhalation, or cutaneous pathways, by means of direct or indirect contact with the product [5]. BPA is considered to be an xenoestrogen or an endocrine disruptor, capable of acting in the animal organism and altering endocrine functions. It does so by mimicking or blocking endogenous hormones, acting as an estrogen receptor agonist, having a similar effect to 17β-estradiol, and altering the production of thyroid hormones [2,6,7]. In mammals, the metabolization of BPA in the organism after its ingestion is done by the liver [5], and in fish, in addition to in the liver, it can also occur in the intestine, as has been evidenced in carp (*Cyprinus carpino*) [8].

The zebrafish (*Danio rerio*) is a teleost that has been much used in research for the past few decades. Due to the similarity of its genome to the human one, its capacity to reproduce during the whole year, its small size, and low maintenance cost, it has become a good experimental model for genetic and toxicology studies [9,10]. In addition, its high sensitivity to chemical substances in water permits its use in studies assessing the toxicity and teratogenicity of endocrine-disrupting substances such as BPA [11,12]. The employment of zebrafish embryos in toxicity assays is also considered to be an alternative to the use of animal models; zebrafish embryotoxicity tests (ZET) [13] and/or the acute toxicity test in fish embryos (FET) can be a predictive model for standard tests on mammals for developmental prenatal toxicity [14]. In addition, there is an ongoing concern about the effects of endocrine disruptors on subsequent generations. Thus, a separate one-generation extended generation reproduction test specifically for zebrafish (ZEOGRT) is being currently standardized by the Organization for Economic Cooperation and Development (OECD) [15]. Those tests, based on zebrafish, are also employed for the identification of the teratogenic effects of a certain substance in embryos. The final points in that test determine, in embryos and larvae, signs of developmental toxicity, as well as the presence or absence of characteristic indicators that should be evaluated at each stage, such as the embryo’s morphology, somite formation, embryonic movement, and heartbeat, in addition to observing possible teratogenic alterations at a cephalic, caudal, cardiac, and/or morphological level [13,16,17].

There is some evidence that exposure to endocrine disruptors could selectively activate or inactivate parts of the genome, causing epigenetic effects and triggering alterations in reproduction and in the coming generations [18,19,20]. Therefore, teratogenicity studies are necessary to complete those on endocrine disruptors. In 2020, the European Union added another amendment to Directive 98/83/EC concerning the quality of water for human consumption with the new Directive (EU) 2020/2184, in which the acceptable concentration of endocrine-disrupting substances, microplastics, including BPA, was reduced. The main focus of the European Food Safety Authority (EFSA) since 2015 has especially been on BPA, which was added to this Directive with a health-based parametric value of 2.5 µg L^−1^ in water [21,22]. Considering the risk of being exposed to the disruptors, this study aims to determine the changes caused by BPA in ovaries and testicles of adult fish exposed to it, as well as assessing the possible alterations that could be caused by this exposure in the embryonic development of generation F1.

## 2. Materials and Methods

### 2.1. Substance Preparation

BPA 99%, 2,2-bis (4-hydroxyphenyl) propane (Sigma^®^ Aldrich), was previously mixed with ethanol and diluted in distilled water for the preparation of a base solution. Next, it was added to the aquarium water for the exposure of the adult fish to concentrations of 500, 50, and 5 μg L^−1^ of BPA [23,24,25].

### 2.2. Determination of BPA in the Water and in the Fish

For the evaluation of the BPA concentration during the testing period, samples were taken of 20 mL of water in each aquarium once a week (days 7, 14, and 21 of the exposure). The samples were kept frozen up to the analytical verification of the above concentrations. Prior to the analysis, the samples were defrosted and processed for their conditioning with ammonium hydroxide; then, they were filtered through 0.22 micra and evaluated in a negative ionization LC-MS/MS system with a detection limit of 0.2–0.3 µg L^−1^.

The BPA concentration in fish after 21 days of exposure was also evaluated by means of the individual homogenate of the bodies of 3 females and 3 males, which were randomly selected from each experimental group. The fish samples were homogenized at 10,000 G in 1 mL tubes, at a 1:2 ratio of body weight, in a buffer solution (50 mM Tris-HCl pH 7.4). The samples were subsequently processed for their extraction and purification and finally transferred into vials, using for this purpose a modification of the technique employed by Schmidt et al. (2008) [26]. Then, 20 µL was injected in an LC-MS/MS triple quadrupole detector 1200 L system for the final BPA quantification. The HPLC System conditions were the same as those previously described by our research group [27].

### 2.3. Experiment Groups

A total of 80 healthy adult zebrafish (*Danio rerio*), approximately 28 weeks old, were used. The fish were kept in reproduction aquariums during the 2-week acclimatization period and with a period of exposure to BPA of 21 days. Each aquarium was furnished with internal partitions to prevent the adults coming into contact with the embryos in the hatching. The proportion of males and females per aquarium was of 5/5 [12,28]. The BPA exposure groups were Group 1 (G1) 500 µg L^−1^; Group 2 (G2) 50 µg L^−1^; and Group 3 (G3) 5 µg L^−1^ of BPA, as well as a negative Control group. All of them were carried out in duplicate; i.e., a total of 20 fish per group (*n* = 20). The darkness cycle was of 14/10 h. The water was kept continuously oxygenated. The mean temperature of the water and of the assay room was maintained, respectively, at 26.4 ± 1.1 °C and 25.4 ± 1.1 °C, the pH at 7.4 ± 0.1, with all the water parameters being controlled daily during the acclimatization and exposure periods. The fish were fed twice a day with dry commercial food (Vitality e Color^®^), supplemented with prawn nauplii (*Artemia salina*). All the procedures complied with the instructions of the Animal Experimentation Committee, following the indications of Directive 2010/63/EU [29] and those of the OECD [14,28].

### 2.4. Evaluation of Embryos

The embryos from each aquarium (generation F1) were collected daily during the whole period of the reproducers’ exposure. To maintain them, the embryonic medium E3 (5 mM NaCl; 0.17 mM KCl; 0.33 mM CaCl_2_; 0.33 mM MgSO_4_) [30] was used, without adding BPA. Before transferring them to plates, they were rinsed two or three times, also with E3 medium. Finally, the embryos were placed on Petri dishes, segmented at a proportion of 45/dish (15 per section). They were incubated at a controlled temperature of 27.3 ± 1.1 °C during the evaluation period and inspected visually under a stereoscopic microscope at 24, 48, 72, and 96 h post fertilization (hpf) [11,14,16]. In the first 24 h, the coagulated embryos were discarded from the study. The embryotoxicity indicators contemplated included alterations related to the general development of the embryos. Other more specific ones were the degree of tail detachment, the formation of somites, eye and/or ear development, the body pigmentation level (as from 24 hpf), the presence of heartbeat, establishment of blood circulation, movements (as from 48 hpf), heart alterations (edema, morphological alterations, and arrythmias); cephalic malformations (absence of ocular structures, unequal sizes of the jaw or eyes); skeletal alterations (escoliosis), and edema in the yolk sac [13]. Likewise, the mortality percentage (dead individuals out of the total number of embryos) was evaluated at 24 h, the eclosion percentage (total of larvae out of the number of individuals) was evaluated at 48 hpf, and the embryotoxicity percentage (total number of embryos with embryotoxicity alterations out of the total number of embryos) was evaluated at 24 hpf [11,14,16,31].

### 2.5. Structural and Ultrastructural Histopathological Study

Once the 21-day exposure period was over, the reproducers were sacrificed with an overdose of an anesthetic solution of tricaine methanosulfonate (MS-222^®^ 500 mg L^−1^; Sigma-Aldrich) buffered with bicarbonate of soda (300 mg L^−1^; Sigma-Aldrich), and finally, regulated necropsies were performed. Samples were taken of the gonads (ovaries and testicles) of the 56 adult fish (seven males and seven females per each experimental group). For the histopathological study, the samples were stored in formol (Sigma^®^ Aldrich) buffered at 10%, treated in different concentrations of alcohol and stained with hematoxylin–eosin (HE) for observation through a Leitz Ortholux photomicroscope (Ernst Leitz GMBH Wetzlar).

For electron microscopy, randomly selected gonad samples were first fixed in a 2% glutaraldehyde solution in 0.1 M phosphate buffer (pH 7.4, 4 °C, overnight) and later fixed in 1% osmium tetroxide in 0.1 M phosphate buffer (pH 7.4) for 30 min. After dehydration in a graded ethanol series and embedding in Araldite, semi-thin and ultra-thin sections were cut on an LKB ultramicrotome (LKB). The semi-thin sections were stained with toluidine blue, whereas the ultra-thin sections were double-stained with uranyl acetate and lead citrate. Tissue sections were examined under a JEM 1400 transmission electron microscope (TEM; JEOL, Ltd. Freising, Germany).

### 2.6. Statistical Analysis

All the embryotoxicity alterations were compiled in Excel templates for the quantitative analysis, with a calculation of their percentages in the different evaluation groups [16]. The embryotoxicity percentage, at each hour post-fertilization, was reckoned by the proportion of embryos and larvae altered out of the number of embryos alive at 24 h. Similarly, the percentage of coagulated embryos was calculated by taking the proportion of the latter out of the total number of embryos collected in each group before 24 hpf. In addition, the mortality and hatching percentages were evaluated at each observation time, calculating them based on the total number of embryos and/or larvae in each one. For the graph of the embryotoxicity indicators, the program *GraphPadPrism* version 9.0.0 [32] was used by applying the Kruskal–Wallis test once the data did not follow a normal distribution, considering statistically significant differences between the groups studied with a value of *p <* 0.05.

For the quantitative and statistical analyses of the principal histopathological alterations, the findings were typified in different categories in terms of the variable analyzed. The Chi-square test was used to verify the degree of correlation between the lesions found and the BPA doses administered. The comparison of proportions between doses was also applied to determine the significant differences between the different groups by considering *p <* 0.05 as being a significant value.

## 3. Results

### 3.1. BPA in the Water and in the Fish

The BPA concentrations found in the water of the different exposure aquariums are shown in Table 1. Those values are very close to those proposed for each exposure group and are absent in the control one.

In the evaluation of the BPA concentration in the fish, values of 14.781 ± 0.026 μg/g in G1; 0.285 ± 0.0013 μg/g in G2; and 0.035 ± 0.004 μg/g in G3, were found, and none were detected in the control group fish.

### 3.2. Embryo Evaluation

**Figure 1:**
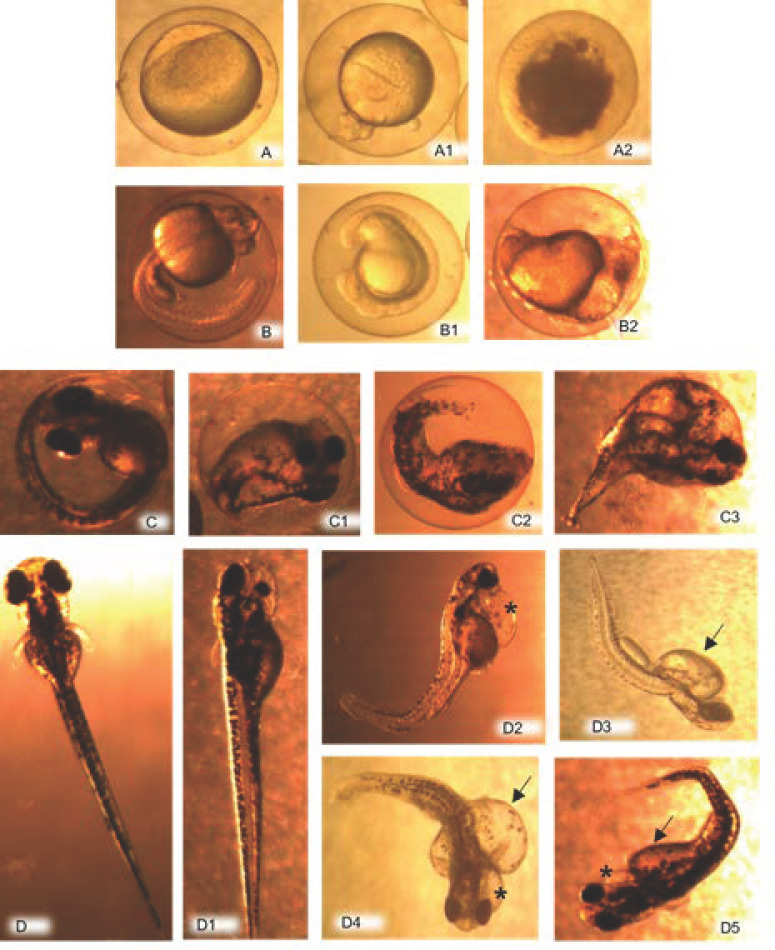
Apparently normal embryos and larvae (control group) and with characteristic indicators of embryotoxicity, proceeding from groups treated with BPA. Embryos (**A**) (≤1 hpf), (**B**) (24 hpf), (**C**) (48 hpf), and larva (**D**) (72 hpf) apparently normal (control group); Embryos of G3 (**A1**,**A2**) with signs of coagulation (24 hpf); Embryo of G3 (**B1**) with no tail detachment (24 hpf); Embryo of G2 (**B2**) with musculoskeletal malformation and non-formation of somites (24 hpf); Embryos (C1,C3) of G1 and (**C2**) of G2 with musculoskeletal malformations, edema in yolk sac, heart and cephalic malformations (at between 48 and 72 hpf); Larvae of G1 (**D1**,**D4**) cephalic malformations (at between 72 and 96 hpf), where the larva in image D1 shows the discrepant formation of the eyes; Larvae (**D2**) of G2, (**D4**,**D5**) of G1 with subendocardial edema (*) (at between 72 and 96 hpf); Larvae of G2 (**D3**) and G1 (**D4**,**D5**) with edema in yolk sac (arrows) (at between 72 and 96 hpf); Larvae (**D3**,**D5**) with musculoskeletal alterations (at between 72 and 96 hpf).

During the first 24 hpf, the embryos with signs of coagulation were discarded (Figure 1(A1,A2). The mean of the coagulates had varied from 378.0 ± 251.7 (58% of the total) eggs in G2 and 418.0 ± 161.2 (56% of the total) in the control group. The statistical analysis did not give any significant differences between groups.

**Figure 2:**
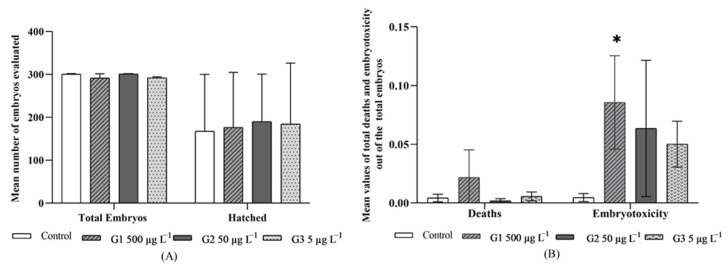
(**A**) Mean of total embryos evaluated and hatching observed; (**B**) Average mortality (dead out of the total embryos or larvae evaluated) and mean embryotoxicity (total embryos or larvae evaluated with embryotoxicity indicators) in the Groups Control, G1, G2, and G3 at between 24 and 96 hpf. The top lines show the standard deviation and the symbol (*) indicates the statistical difference of G1 for the control group in the Kruskal–Wallis (*p* = 0.0405) test for the mean embryotoxicity (total of embryos or larvae with embryotoxicity indicators).

The embryos viable at 24 hpf in each of the groups were evaluated for signs of embryotoxicity, such as mortality, hatching, and specific malformation alterations. The total mean of viable embryos per group at 24 hpf was of 300.0 ± 113.1 embryos in G1; 301.5 ± 242.5 in G2; 294.0 ± 31.1 in G3 and 302.0 ± 113.1 in the control group (Figure 2).

In the mortality evaluation, the absence of a heartbeat, the incorrect establishment of the bloodstream, and the lack of movements were observed. The mortality percentage (embryonic mortality out of the total embryos evaluated) was generally low as from 24 hpf. However, in comparing the different exposure groups, it was considerably higher in embryos from the G1 (500 µg L^−1^) at all the times evaluated (Figure 2B), with a mean mortality of 0.02 ± 0.02 and reaching the highest value at 48 hpf with a mortality percentage of around 5% (Figure 2B).

Another evaluation point was the hatching of the embryos that also helped to interpret BPA toxicity signs. Hatching, in all the exposure groups, began at 48 hpf, with the percentage of hatching (total number of larvae out of total embryos) at this time of 48 hpf being higher in the embryos proceeding from the group exposed to 50 µg L^−1^ (G2), with a corresponding percentage of 22.7% of hatching. However, at 72 and 96 hpf, the hatching percentage was higher (86.4 and 96.7%, respectively) in the group exposed to the lowest concentration (G3), with a mean hatching of 0.63 ± 0.49, this being very close to that of group G2 (0.63 ± 0.37). In addition, the control and G1 groups had a mean hatching of 0.56 ± 0.44 and 0.62 ± 0.46, respectively, with no statistically significant differences being found between any of the groups evaluated (Figure 2A). In the evaluation of embryos for indicators of embryotoxicity, plus links to malformations (non-detachment of the tail, lack of somites, absence of heartbeat, skeletal and cardiac malformations, edema in yolk sac), the exposed groups were the affected ones. In a general assessment, G1 gave, at all the observation times, the highest embryotoxicity percentages (total of embryos and/or larvae with embryotoxicity indicators out of the total of embryos evaluated), in relation to the rest of the groups exposed. The mean of embryotoxicity in G1 was higher, 0.09 ± 0.04, than the means of the other groups, and it was statistically significant (*p* < 0.05) in comparison with the control group, which gave a mean embryotoxicity of 0.004 ± 0.003 (Figure 2B), which was too low.

With respect to the embryotoxicity indicators, in a separate evaluation of each alteration found, those that refer to the non-formation of somites and delayed absence of tail detachment (Figure 1(B1,B2)) were predominant in G1, with a total of 50% of the individuals with those embryotoxicity signs at the time of 24 hpf and with 21% of embryos affected at 48 hpf. The control group did not present any such alterations.

Cephalic malformations were found in all the groups treated, with the exception of the control one, with a higher percentage in G1 at 96 hpf when 29% of the total embryos were altered (Figure 1(D1)). In relation to cardiac alterations (Figure 1(D2,D4,D5), these were observed more frequently in group G1 individuals, being most apparent at 72 hpf, when they were seen in 69% of the total of individuals that had some type of malformation. The anomalies at a skeletal level (Figure 1(C1–C3)) were evidenced in individuals from all the exposure groups, with a greater incidence in G1 at 48 and 72 hpf (46% and 41%, respectively, out of all the individuals with malformations), which were followed by G3 at 96 hpf (41% of the total of embryos with malformations). The high incidence of the presence of edemas in the yolk sac found is worth noting (Figure 1(D2–D5)). This anomaly presents itself with greater frequency in G1 at 96 hpf, when 93% of all the embryos with malformations displayed it. Likewise, in G2, at 48 and 72 hpf, this alteration was observed in 92% and 82%, respectively, of the embryos with malformations recorded. None of the groups evaluated demonstrated any lack of body pigmentation or the absence of the ocular sphere or of ears.

### 3.3. Structural and Ultrastructural Histopathological Study

**Figure 3:**
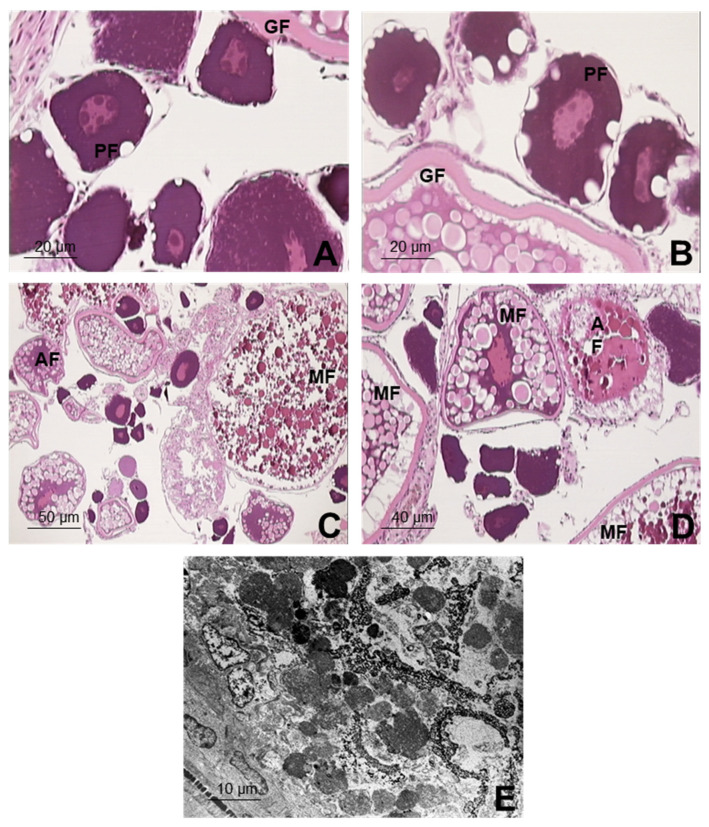
Ovarian parenchyma in zebrafish exposed for 21 days to BPA, plus the control group, evaluated under optical and electron microscopes: (**A**) Control group: Normal ovarian parenchyma, with numerous primordial follicles. Detail of primary follicle (PF) and growing follicle (GF); (**B**) Group 3 (5 µg L^−1^): Diverse ovarian follicles; primary follicle (PF) with highly vacuolized cytoplasm; growing follicles (GF) with a nucleus with rough edges; (**C**) Group 2 (50 µg L^−1^): Diverse ovarian follicles; growing follicles with a highly developed pellucida membrane, a mature follicle (MF), and an atretic follicle (AF); (**D**) Group 1 (500 µg L^−1^): Numerous atretic follicles (AF); the mature follicles (MF) show abundant granulations and lipidic droplets; (**E**) Under an electron microscope (Group 1): cytoplasmic rests of the degenerated atretic oocyte.

In the micro and ultrastructural histopathological study of the ovary in the control group, no alterations were observed in the ovarian parenchyma. It exhibited an extensive growth of follicles with a correct distribution of all their elements. The normal primordial follicle was observed as being integrated by the oocyte with a large spherical nucleus and a cytoplasm with highly intense basophilia. The growing follicles were formed by the oocyte, entirely surrounded by a thick acidophilus hairy membrane, with the nuclei being maintained in the center of the oocyte. Finally, the mature follicles represented the oocytes that, in their final phase, are released to the outside. In those follicles, a masking of the nucleus is produced, so that the cytoplasm can be especially highlighted. It is large in size and is occupied practically entirely by large yolk granules and lipid vacuoles (Figure 3A).

In G3 (5 µg L^−1^), the same types of follicles described in the control group were found. All of them kept up the composition and type of lining cells with no evidence of alterations either under the optical or the electron microscopes. There were few atretic follicles in this exposure group, but they were not statistically different from those of the control group (Figure 3B). For G2 (50 µg L^−1^), the cytoplasm of the primary follicles was found to be vacuolized (Figure 3C). In the growing follicles, there was a slight increase in vesicles and lipid droplets, with a statistical difference compared to the G3 and control group (*p* < 0.05). In the latter, it is worth noting that the nucleus had rough edges. In general, in G2, the degradation of follicle components was considered to be moderate, and it was observed in most of the females evaluated for this concentration of BPA (71.4%), with a significant difference for the other groups exposed to lower concentrations (*p* < 0.05). There was a notable increase in atretic follicles with a moderate disintegration of their components (57.1% of the ovaries observed), which was also observed under the electron microscope. In the group exposed to the highest concentration of BPA (500 µg L^−1^), G1, both the growing follicles and the mature ones exhibited an important development, with abundant granules and lipidic droplets, as well as numerous atretic follicles with a degradation of all the components of the ovarian follicle (Figure 3D). These alterations presented high severity with significant statistical differences in relation to the other groups (*p* < 0.05). In G1, we can also highlight an interstitial fibrosis, which is characterized by the presence of fibrous connective tissue inside the ovarian stroma, with granulomatose infiltrations only observed in this group; significant differences were found with the rest of the groups (*p* < 0.05). In addition, noted under the electron microscope in G1, there was a significantly high increase in vacuolizations in the cytoplasm, greatly destructured nuclei, and extensive degenerative processes, in which there were abundant atretic follicles with a severe degradation of all their components (*p* < 0.05) (Figure 3E).

**Figure 4:**
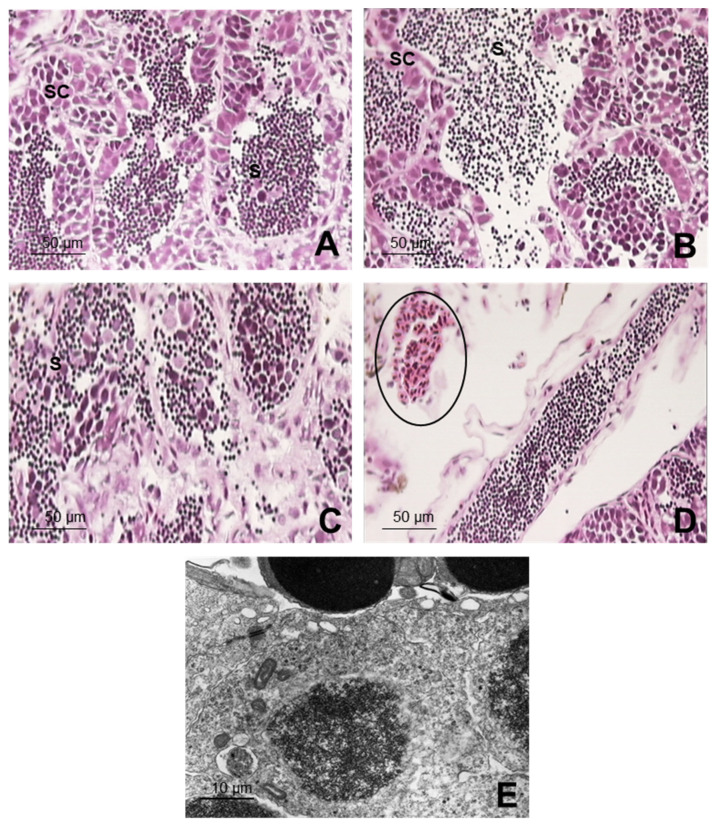
Testicular parenchyma of zebrafish exposed during 21 days to BPA plus the control group, as evaluated by optical and electron microscopes. Optical microscope. (**A**) Control group: cell components forming the spermatogenesis, as well as the Sertoli cells (SC), abundant spermatozoa (S) in the spermatic cord lights; (**B**) Group 3 (5 µg L^−1^): swelling was noted in the Sertoli cells (SC), together with the release of sperm (S) due to discontinuity in the testicular cords; (**C**) Group 2 (50 µg L^−1^): cell loss with a drastic diminution in the number of spermatozoa (S), cytoplasmic vacuolizations and disorganization in the testicular cords; (**D**) Group 1(500 µg L^−1^): highly degenerated parenchyma, edematose and hemorrhagic (circle). (**E**) Under electron microscope (Group 1): Sertoli cell in testicular parenchyma with swelling and hypertrophy.

In the structural and microstructural evaluation of the testicles, the testicular parenchyma of the zebrafish corresponding to the control group (Figure 4A) was seen to be organized in many well-developed seminiferous tubules, displaying all the cell components with a normal distribution, nor did the Sertoli cells present any alteration. The spermatozoa in their different developmental phases also had a normal aspect, although some of them had heads that were more oval-shaped and not so spherical, in which a very dense nucleus with a cavity stood out and in which a centriole was integrated. The latter was constituted in the basal body of an extremely long, type 9 + 2, cilium. The cytoplasm hardly existed, and there was only a protuberance in which some mitochondria were grouped, related to the cilium, and a thin, laminar projection that was initiated in the ciliary base.

In G3, the testicular parenchyma showed mild signs of cell component degeneration in 85.7% of the individuals evaluated, which differed significantly from the control (*p* < 0.05). There was swelling in the Sertoli cells and a partial loss of spermatogonia-producing cells. Although the number of spermatozoa was maintained, there was discontinuity in the testicular cords, so that some spermatozoa were released into the inside of the testicle (Figure 4B). These alterations were also manifested under the electron microscope, in which mild signs of cell component degeneration were observed, but the rest were very similar to those of the control group. The testicles in G2 presented moderate signs of degeneration (Figure 4C), showing clearly significant differences compared to those seen in lower concentration groups (*p* <0.05). A cell loss can be highlighted, with a drastic diminution in the number of spermatozoa, cytoplasmic vacuolizations, and a disorganization of the testicle sacs. All these alterations were produced in all the cell components and were observed in a high percentage of males evaluated. The altered cell components were also observed under the electron microscope, with the degenerated Sertoli cells presenting a vacuolization of the cytoplasm; degeneration and swelling in the spermatogonias; together with degradation of the spermatocyte chromatin, nuclei, and implementation pits in the spermatids. In addition, the spermatozoa had immature, degenerated shapes. For all these alterations, significant differences were found compared to those individuals exposed to lower concentrations as well as to the control group (*p* < 0.05). Group G1 (500 µg L^−1^) exhibited a parenchyma with severe alterations, such as cell degeneration, highly marked edema, and hemorrhage processes in all the males evaluated (Figure 4D), with statistically significant differences compared to G3 and the control group (*p* < 0.05). Under the electron microscope, these findings were recorded with a similar frequency and intensity. In general, cell degenerations were observed as well as a very significant increase in Sertoli cells and a morphology of active ones, with spermatogonias diminished in number and generated. Although they were altered, the spermatocytes maintained their meiotic divisions. Vacuolization processes of the spermatids were noted both in their cytoplasm and in their nuclear envelope. The spermatozoa were highly reduced in numbers: some with a normal morphology and others with degenerative processes, i.e., swelling and hypertrophy and monstrous shapes (Figure 4E). Significant differences were found for all these alterations compared to what was described for G3 and the control group (*p* < 0.05).

## 4. Discussion

The animals were exposed to BPA doses that were considered to be low and below the lethal dose, under 1000 µg L^−1^ BPA in water [23]. The BPA values were very close to the expected values in water for each of the experimental groups. Although the concentrations found in the fish were relatively numerically low, the doses employed were enough to trigger alterations in the embryos and in the adults.

Endocrine disruptors can activate, repress, or interfere in the synthesis and metabolism of hormones. BPA, currently a very well-known endocrine disruptor, possesses the agonistic ability of estrogen receptors and is also able to antagonize them in tissues such as the brain and in the reproductive organs of humans, rat, mouse, and fish such as zebrafish [23,33,34,35]. As no alterations were found in any of the growth stages of the zebrafish control group, a larger number of altered embryos could be associated with the BPA exposure groups (G1, G2, and G3). Those alterations can be explained by the estrogenic action of the compound and the reinforcement of the effects on the thyroid hormone of the growing fish. In this regard, BPA acts by causing the altered transcription of different genes sensitive to T3, which are biological markers related to the thyroid hormone function in skeletal and bone growth as well as eye and hemopoietic development [36]. In addition, with respect to the higher mortality observed, although this had already been described in embryos directly exposed to concentrations of 5000 to 20,000 µg L^−1^ for several hours [17], in our study, it was observed that the action of BPA in much lower concentrations such as those used (approximately 500, 50, and 5 µg L^−1^), in which, besides, the embryos were not exposed for longer than one hour until collected, were sufficient to cause a considerable mortality percentage, mainly in the concentration of 500 µg L^−1^, generating important alterations in growth, basically classified as being teratogenic.

Our results coincide with those of other published studies related to exposure to increasing concentrations of BPA. These included an increase in heart and cephalic malformations, alterations in the swim bladder, formation of edemas in yolk sac, no formation of somites, as well as the non-detachment of the tail [37,38]. These alterations are linked to the deregulation of genes related to the endocrine system (regulator of embryonic growth (Egr2) caused by the BPA, mainly affecting cardiovascular and neurological development [37]. The association between direct embryonic exposure to BPA and the presence of heart and cephalic malformations was also described by other authors [39,40]. The alterations found by us agree with those of those authors despite the embryos in our study not having been in contact with BPA during the evaluation time (96 hpf). This demonstrated that the effects of BPA could be in generation F1, whether or not there has been any direct contact with the chemical. The alteration related to the formation of edemas in the yolk sac could be linked to the interruption in the metabolism of the lipids provoked by the BPA once the yolk sac acts as an organ for reserving nutrients, such as phospholipids and triacyglycerols, for the embryo’s growth. Therefore, the lipid alterations could directly affect the yolk sac. In rats and mice, for example, BPA is able to increase the cell density of the adipocytes and induce the accumulation of hepatic lipids, which reinforces the hypothesis that the metabolism of the lipids is one of the principal pathways of BPA [41]. With respect to the hatching percentage, higher values were obtained in the embryos of progenitors exposed to BPA, with the first records coinciding in all the cases, although at a lower proportion at 48 hpf. The highest hatching percentages were recorded at between 72 and 96 hpf. These results coincide with that expected in relation to the hatching time, which ranged around 72 hpf. However, the data in the literature with respect to this parameter in assays with BPA are controversial, since some studies point to BPA as being a substance capable of causing a delay in hatching at 72 hpf in zebrafish [38], and others did not find any apparent differences between the groups evaluated [41]. BPA accumulation in rainbow trout eggs, prior to fertilization, did not modify their fertilization rate but delayed hatching and larval growth compared to controls [42]. In a study similar to the one we conducted, parental exposure to BPS (BPA analogues) resulted in delayed hatching of the F1 generation of zebrafish [25], but this was not significantly evidenced in our study with BPA.

Those developmental alterations following exposure of the reproductive tract are reflected in the development of the next generation. In humans, exposure to higher doses of BPA than those used in this study (concentrations relevant to human health) can lead to earlier puberty in individuals exposed during the fetal phase and increased prostate growth in humans [43], which is attributed to the known epigenetic effects of endocrine-disrupting substances [44]. In studies assessing the fecundity of other zebrafish generations, adverse transgenerational effects on fecundity were also observed in the offspring of fish exposed to BPA [20].

With respect to the histopathology studies, the characteristic of BPA being a selective modulator of the estrogen receptors α and β, as commented on above, should be considered. Its estrogenic activity acts directly on the vitellogenin, which is a protein secreted by hepatocytes in the liver that is synthesized in response to endogenous estrogens, such as 17β-estradiol (E2), released into the bloodstream, and transported to the ovaries, acting in the development of the oocyte [24,36,45]. BPA as an endocrine disruptor can significantly increase vitellogenin transcription [36], thus being capable of causing negative effects on the function and development of reproductive and neuronal systems [46]. Female mice exposed prematurely to BPA manifested a reduction in fertility and fecundity [47]. It can also alter the morphology and functionality of the reproductive tract in females as well as affect the development of the mammary gland at puberty and compromise sexual differentiation in the brain [48,49,50]. The BPA concentrations used in the experiment groups are in the range considered to be a low dose for aquatic animals, which is under 1000 µg L^−1^ of BPA in water [23]. Despite the low dose used, significant alterations were found in our study, both under the optical and the electron microscopes, these being more apparent in the individuals exposed to higher concentrations of BPA (500 µg L^−1^).

One of the alterations found in the histopathological study, both of group G1 and of G2, was the presence of numerous lipidic vesicles (granules and lipidic droplets) in their cytoplasm. In this sense, it should be considered that the lipids found naturally in the eggs of fish are derived from the fatty acid of their diet, from the reserves, and/or are synthesized by the animal’s organism [51]. In addition, the lipids reach the oocytes by means of the vitellogenin, which is rich in polar lipids, and probably by other lipoproteins especially lipoproteic ones of a low density that are rich in triaglycerol, which will help to supply energy for the embryonic and larval development [51]. This means that the lipidic composition of the oocytes is directly related to the concentration of vitellogenin in the blood, mainly during the vitellogenesis phase, in which the egg yolk is produced [52]. As the alterations found were more accentuated in the groups with a higher BPA concentration, its toxic action in the female zebrafish gonads exposed can be directly associated. Once the estrogenic action of BPA has influenced the concentration of vitellogenin upwardly, BPA can also be associated with the occurrence of numerous atretic follicles and the degradation of all the follicle components, with both these alterations being more frequent and apparent in the groups treated with BPA. These results coincide with those published by other authors [27,53]. Disruption of porcine oocyte maturation following BPA treatment was evidenced in in vitro studies, which was mainly due to alterations in the oocyte cytoskeleton, epigenetic modification, oxidative stress, autophagy, and oocyte apoptosis, compromising porcine reproductive systems [54]. In addition, the follicular atresia process observed in groups exposed to BPA has been described in individuals in which there is no egg-laying and the ooctye is re-absorbed by the animal’s organism, which increases in cultivated or wild populations submitted to high levels of exploitation and capture, possibly being originated by different dysfunctions or reproductive alterations [55,56].

Alterations were observed in the structural and microstructural evaluation of the males, such as parenchyma degeneration, Sertoli cell swelling, and a diminution in the number of spermatozoa; the latter became proportionally aggravated the higher the BPA concentrations used. In the highest concentration, 500 µg L^−1^ in G1, even more serious alterations were found, such as edema and hemorrhage in the parenchyma, diminution in spermatogonias, increase in Sertoli cells that remained active, a reduction in spermatozoa, and alterations in their morphology. Those alterations could be due to the increase in estrogenic actions once the BPA can act as 17β-estradiol (E2). The E2 is normally present in low concentrations in the blood of males, acting importantly in the genic expression of the testicles [57,58]. The increase in E2 in males can be confirmed by the concomitant increase in the vitellogenin in their blood once the E2 has the capacity to induce the vitellogenin coding gene between 10 and 30 times. In addition, exposure to high levels of estrogens can reduce the volume of seminal liquid, increase spermatozoid density, and lead to infertility [58]. BPA can also act as a steroid receptor [2,6], which is associated with the testicle maturation process and spermatogenesis. The sperm alterations evidenced in this study corroborate the alterations caused by BPA in some fish species of brown trout (*Salmo trutta*) and *Pimephales promelas*, in which there was a reduction in sperm density and motility [59] and in sperm production [60]. The severe reduction in sperm count found in our study confirms the findings from similar studies of several zebrafish offspring following BPA exposure [18,36,59]. Most of these changes in maturation in the genic expression seem to be highly correlated with the changes in the levels of circulating androgens [58], which is another indicator of the fact that the presence of BPA may be altering spermatogenesis negatively. The males were seen to be extremely sensitive to BPA since, starting from the group with the lowest concentration (G3–5 µg L^−1^), structural alterations in their testicles were already observed. The seriousness of those alterations was directly related to the increase in BPA in the groups.

## 5. Conclusions

This study demonstrates that in the F1 generation of embryos of breeders exposed to BPA, alterations in their growth classified as embryotoxicity indicators were observed. These malformations were more frequent in individuals from the group exposed to the highest concentration (500 µg L^−1^). Among the malformations with the greatest incidence noted, those standing out were the absence of tail detachment, the non-formation of somites, cephalic malformations, heart and musculoskeletal alterations, and edema of the yolk sac, the latter being the most common one among the individuals evaluated. Those embryotoxicity indicators could be associated with the toxic and estrogenic action of BPA, as was demonstrated by the low incidence observed in the control group. It was seen that BPA is capable of causing structural and microstructural alterations in the gonads of adult zebrafish after exposure during 21 days. The ovarian alterations perceived were atresia, degeneration and, in testicles, cell degeneration and loss of normal cell structures, which could considerably reduce fertility, both in females and males. In view of the inclusion of specific endpoints of certain endocrine disruptors, this study can contribute useful evidence on the probable causality of transgenerational effects, which is a key issue in the definition of endocrine disruptors.

## Figures and Tables

**Table 1:** Mean and deviation of the BPA concentrations in the aquarium water of each experimental group.

BPA Concentration in Water (µg L^−1^)				
Exposure Days	G1 (500 µg L^−1)^	G2 (50 µg L^−1^)	G3 (5 µg L^−1^)	Control
7	484.871 ± 0.001	47.312 ± 0.003	4.545 ± 0.005	nd *
14	482.442 ± 0.004	48.449 ± 0.012	4.694 ± 0.008	nd *
21	480.757 ± 0.002	47.357 ± 0.008	4.753 ± 0.011	nd *

* nd = no detected.

## Data Availability

The data that support the findings of this study are available on request from the corresponding author.
